# Hepatitis B virus (HBV)-specific T-cell responses to recombinant HBV core protein in patients with normal liver function and co-infected with chronic HBV and human immunodeficiency virus 1 (HIV-1)

**DOI:** 10.1186/1743-422X-10-232

**Published:** 2013-07-12

**Authors:** Xin Zhang, Hanqian Xing, Xia Feng, Haiping Zhang, Yi Wang, Huiping Yan

**Affiliations:** 1 Infection and Immunity Research center of Beijing Youan Hospital, Capital Medical University, Beijing, China100069; 2 Liver Failure Treatment & Research center, 302 Military Hospital, Beijing 100039, China

## Abstract

**Background:**

Little is known about HBV-specific T-cell responses in chronic Hepatitis B patients (HBV) that are co-infected with Human immunodeficiency virus type 1 (HIV-1), especially those with normal alanine aminotransferase (ALT) levels.

**Methods:**

Twenty-five patients with chronic HBV (11 hepatitis B e antigen [HBeAg]-positive, 14 HBeAg-negative) were enrolled in a cross-sectional study. A longitudinal study as also conducted in which follow-up was done at 3, 12, and 24 months, after acute HIV-1 infection, in 11 individuals who also had chronic HBV. Peripheral blood mononuclear cells were stimulated with recombinant HBV surface protein (S protein), core protein (C protein) or gag peptide. IFN-γ-secreting T cells were identified by ELISPOT assay.

**Results:**

In the cross-sectional study, co-infected chronic HBV patients had lower C protein-specific T-cell responses compared with mono-infected individuals, though the difference was not significant. In co-infected, chronic HBV patients, the magnitude of C protein-specific T-cell responses was significantly greater in HBeAg-positive subjects compared to HBeAg-negative subjects (p = 0.011). C protein-specific T-cell responses were positively correlated with HBV viral load (r_s_ = 0.40, p = 0.046). However, gag-specific T-cell responses were negatively correlated with HIV viral load (r_s_ = −0.44, p = 0.026) and positively correlated with CD4^+^ count (r_s_ = 0.46, p = 0.021). The results were different in mono-infected individuals. PBMCs from co-infected HBeAg-positive patients secreted more specific-IFN**-**γ in cultured supernatants compared with PBMCs from co-infected HBeAg-negative patients (p = 0.019). In the longitudinal study, S protein- and C protein-specific T-cell responses were decreased as the length of follow-up increased (p = 0.034, for S protein; p = 0.105, for C protein). Additionally, the S protein- and C protein-specific T-cell responses were significantly higher in HBeAg-positive patients than in HBeAg-negative patients at 3 and 12 months after HIV-1 infection (all p < 0.05), but not at 24 months. A positive correlation (trend) was found between C protein-specific T-cell responses and HBV viral load at 3 and 12 months after HIV-1 infection.

**Conclusions:**

HBV-specific T-cell responses to recombinant HBV core protein were reduced in chronic HBV patients co-infected with HIV-1. The reduced C protein-specific T cell responses were positively correlated with HBV viral load in co-infected, chronic HBV patients.

## Background

Patients with Human immunodeficiency virus type 1 (HIV-1) are frequently found to be co-infected with Hepatitis B virus (HBV). These patients often have a high prevalence of hepatitis B surface antigen (HBsAg)- or hepatitis B core antibody (HBcAb)-positive HBV serological markers due to common infection routes. Worldwide, it is estimated that 70-90% of HIV-1 patients have evidence of previous HBV infection and 5-15% of HIV-1 patients have current HBV infection and are HBsAg positive. HBV individuals that are co-infected with HIV seroconvert from hepatitis B precore antigen (HBeAg) to anti-HBeAg antibody (HBeAb) less frequently, have higher HBV DNA levels, lower levels of alanine aminotransferase (ALT) and a history of milder necroinflammatory activity, compared to those infected with HBV only [1-4]. In the presence of HIV-1, the progression of HBV related liver disease is accelerated and liver related mortality is significantly increased [5,6]. Additionally, there is an increased risk of persistent chronic infection in individuals infected with HIV-1 who are subsequently infected with HBV [7]. Previous studies have also shown a relatively high prevalence of occult Hepatitis B in HIV-1-infected patients, especially after the withdrawal of antiretroviral therapy. Therefore, determining the pathogenesis associated with HIV-1 and HBV interaction, including the modification of the immune responses, will allow for the development of a rational approach for the management of co-infected individuals.

In persistent HBV monoinfection, there is a reduction in HBV-specific CD4^+^ and CD8^+^ T cells compared with individuals that have successfully cleared the infection. It has been suggested that HBeAg plays a role in facilitating HBV persistence by depleting HBeAg- and HBcAg-specific Th1 CD4^+^ T cells [8]. The reduction in antigen burden following anti-HBV treatment may reduce T cell tolerance and exhaustion, allowing for a more efficient HBV-specific T-cell and B-cell immune response against either HBeAg and/or HBsAg [9]. Many previous studies have focused on the alteration of immune responses in HIV/HBV patients with abnormal liver function and lower CD4 counts, however little is known about individuals with milder liver disease that have normal levels of ALT and higher CD4 counts. In the present study, we examined whether HBV-specific T-cell responses in chronic HBV patients could be influenced by the presence or absence of HBeAg or by the level of HBV DNA, in the presence of HIV-1. Recombinant S and C proteins were used as a stimulus, in both cross-sectional and longitudinal studies, to assess changes in the HBV-specific total T-cell responses in chronic HBV patients with HIV-1 co-infection.

## Results

### Patient characteristics

In cross-sectional study, demographic and clinical details of 25 co-infected chronic HBV patients and 16 mono-infected chronic HBV patients are summarized in Table 1. All co-infected chronic HBV patients had normal liver function, as determined by ALT, AST, and TBIL analysis, within two years of observation. The HIV/HBV co-infected patients mainly included high-risk men-who-have-sex-with-men (MSM). The HBV viral load in the co-infected, chronic HBV patients was comparable to that of the mono-infected, chronic HBV patients. HBV viral load was not correlated with HIV viral load in co-infected, chronic HBV cases. Demographic and clinical details of patients with previous HBV infection, who were co-infected with HIV (n = 14) and who were not co-infected with HIV (n = 16), are summarized in Table 2. HIV patients that had previous HBV infection mainly included high-risk MSM, and had normal liver function, as determined by ALT, AST, TBIL analysis, within two years of observation. The HBV viral load in co-infected patients that had been previously infected with HBV was comparable to that of mono-infected patients that had been previously infected with HBV.

**Table 1:** Demographics of chronic HBV patients

**Parameter**	**Single HBV infection**			**HIV/HBV coinfection**		
	**HBeAg**^**+**^	**HBeAg**^**-**^	**P value**	**HBeAg**^**+**^	**HBeAg**^**-**^	**P value**
No. of Subjects	8	8		11	14	
Gender(no. male/female)	7/1	6/2	NS^a^	11/0	13/1	NS^a^
Age [median (range)] (yr)	36 (22-60)	38 (25-67)	NS^b^	34 (23-40)	32 (25-56)	NS^b^
ALT [median (range)] (U/L)	19.2 (6.1-53)^c^	23.7 (12.2-40)	NS^b^	22.4 (13.5-58)^c^	19.2 (13.7-38)	NS^b^
HBV DNA^d^ [median (range)]	7.71 (0-9.04)	1.04 (0-4.32)	<0.01	6.85 (0-8.51)	1.35 (0-8.98)	<0.01
HIV RNA^d^ [median (range)]	NA	NA		4.8 (0-5.4)	4.98 (0-5.9)	NS^b^
CD4 count [median (range)] (cells/ul)	NA	NA		452 (211-862)	332 (122-704)	NS^b^

*NS* not significant, *NA* not applicable, ^a^Fisher’s exact test; ^b^Mann-Whitney U test; ^c^only one patient had an ALT >40 U/L; ^d^log_10_, ^+^postive; ^-^negtive.

**Table 2:** Demographics of individuals previously infected with HBV

**Parameter**	**Without HIV coinfection**	**With HIV coinfection**	**P value**
No. of patients	16	14	
Gender (no. male/female)	9/7	13/1	0.039^a^
Age			
[median(range)] (yr)	31 (23-46)	33 (23-54)	NS^b^
ALT			
[median(range)] (U/L)	13(4-30)	15.5 (3-50)^c^	NS^b^
HBV DNA^d^			
[median(range)]	<500	<500	NS^b^
HIV RNA^d^			
[median(range)]	NA	4.45 (0-4.93)	NA
CD4 T cell count			
[median(range)] (cells/ul)	NA	323 (76-640)	NA

*NS* not significant, *NA* not applicable, ^a^Fisher’s exact test; ^b^Mann-Whitney U test; ^c^only one patient had an ALT >40 U/L; ^d^log_10_.

In the longitudinal study, 11 individuals with acute HIV-1 and chronic HBV infection had normal levels of ALT, AST and TBIL during the 2-year follow-up (Subject demographic and clinical details are summarized in Table 3).

**Table 3:** Demographics of individuals with chronic HBV and acute HIV-1 co-infection

			**v1**			**v2**			**v3**	
**Patient**	**HBeAg**	**HBV DNA**^**a**^	**HIV RNA**^**a**^	**CD4 count**	**HBV DNA**^**a**^	**HIV RNA**^**a**^	**CD4 count**	**HBV DNA**^**a**^	**HIV RNA**^**a**^	**CD4 count**
AHI01	pos	8.45	5.66	376	7.38	5.66	212	3.52^b^	<ldl^b^	376
AHI02	pos	6.54	4.55	533	7.43	4	298	7.76	ND	405
AHI03	neg	<ldl	3.89	213	<ldl	ND	192	<ldl	ND	189
AHI04	pos	7.71	3.75	345	8.2	4.47	275	8.4	ND	342
AHI05	pos	9.04	5.72	363	8.32	ND	292	7.14	ND	363
AHI06	neg	<ldl	2.23	361	<ldl	ND	345	<ldl	ND	221
AHI07	pos	8.14	6.1	412	8.45	ND	329	7.54	ND	293
AHI08	neg	<ldl	4.92	308	<ldl	4.67	365	<ldl	ND	395
AHI09	neg	<ldl	3.39	922	<ldl	3.3	862	<ldl	ND	692
AHI10	neg	<ldl	6.31	380	<ldl	ND	464	<ldl	ND	243
AHI11	neg	<ldl	5.62	221	<ldl	ND	445	<ldl	ND	445

*pos* HBeAg positive, *neg* HBeAg negative; v1, 2, 3: followed up on 3^rd^, 12^th^, 24^th^ month post acute HIV-1 infection; ^a^log_10_; ^b^HAART with AZT/3TC/LPVr; *ldl* lower limit of detection, *ND* not done.

### In the cross-sectional study, HBV-specific T-cell responses in co-infected individuals were decreased compared to mono-infected individuals

**Figure 1:**
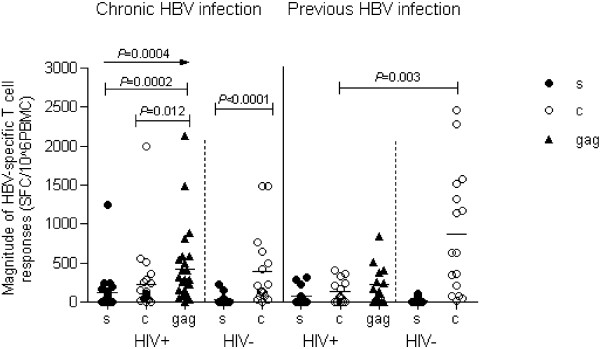
**Magnitude of HBV-specific T-cell responses induced in HBV patients with and without HIV infection.** Left, chronic HBV infection; right, previous HBV infection, HIV+, co-infected with HIV; HIV-, not co-infected with HIV. s: stimulated by S protein; c: stimulated by C protein; gag: stimulated by gag peptide pool.

There was no significant difference in the response frequency to C protein when comparing co-infected, chronic HBV patients and mono-infected, chronic HBV patients (22/25 and 15/16 responded to C-protein, respectively). The magnitude of the C protein induced HBV-specific T-cell responses in co-infected, chronic HBV patients was lower than in mono-infected, chronic HBV patients, however the difference was not statistically significant (p > 0.05; Figure 1 left). On the other hand, in co-infected, chronic HBV patients, gag-specific T cell responses were the highest with all 3 stimuli, including S protein, C protein and gag peptide (p = 0.0004). Gag-specific T cell responses were also higher than S protein- and C protein-specific T cell responses (all p < 0.05; Figure 1 left). In mono-infected, chronic HBV patients, the magnitude of C protein-specific T cell responses was greater than that of S protein-specific T cell responses (p < 0.0001; Figure 1 left).

The response to C protein was less frequent in HIV patients that had previous HBV infection, compared to patients that had previous HBV infection but who did not have HIV (9/14 and 15/16 responded to C protein, respectively; p = 0.072). The magnitude of C protein-specific T-cell responses was significantly weaker in individuals that had been previously infected with HBV and who were co-infected with HIV compared to those who were not co-infected with HIV (p = 0.003; Figure 1 right), however there was no significant difference between these groups in specific T-cell responses to S protein. The median number of total T cells was comparable in co-infected and mono-infected individuals that had previous HBV infection (1346[539–3463]/uL vs. 1361[689–2116]/uL, respectively). Similarly, the total T cell percentage was not significantly different between these two groups. The fact that total T cell number and percentage were not decreased, and that C protein-specific T cell responses were decreased, implies that HBV-specific T cell responses may be impaired in the presence of HIV-1 infection.

### HBV viral load was correlated with the magnitude of HBV-specific T-cell responses in chronic HBV patients co-infected with HIV

**Figure 2:**
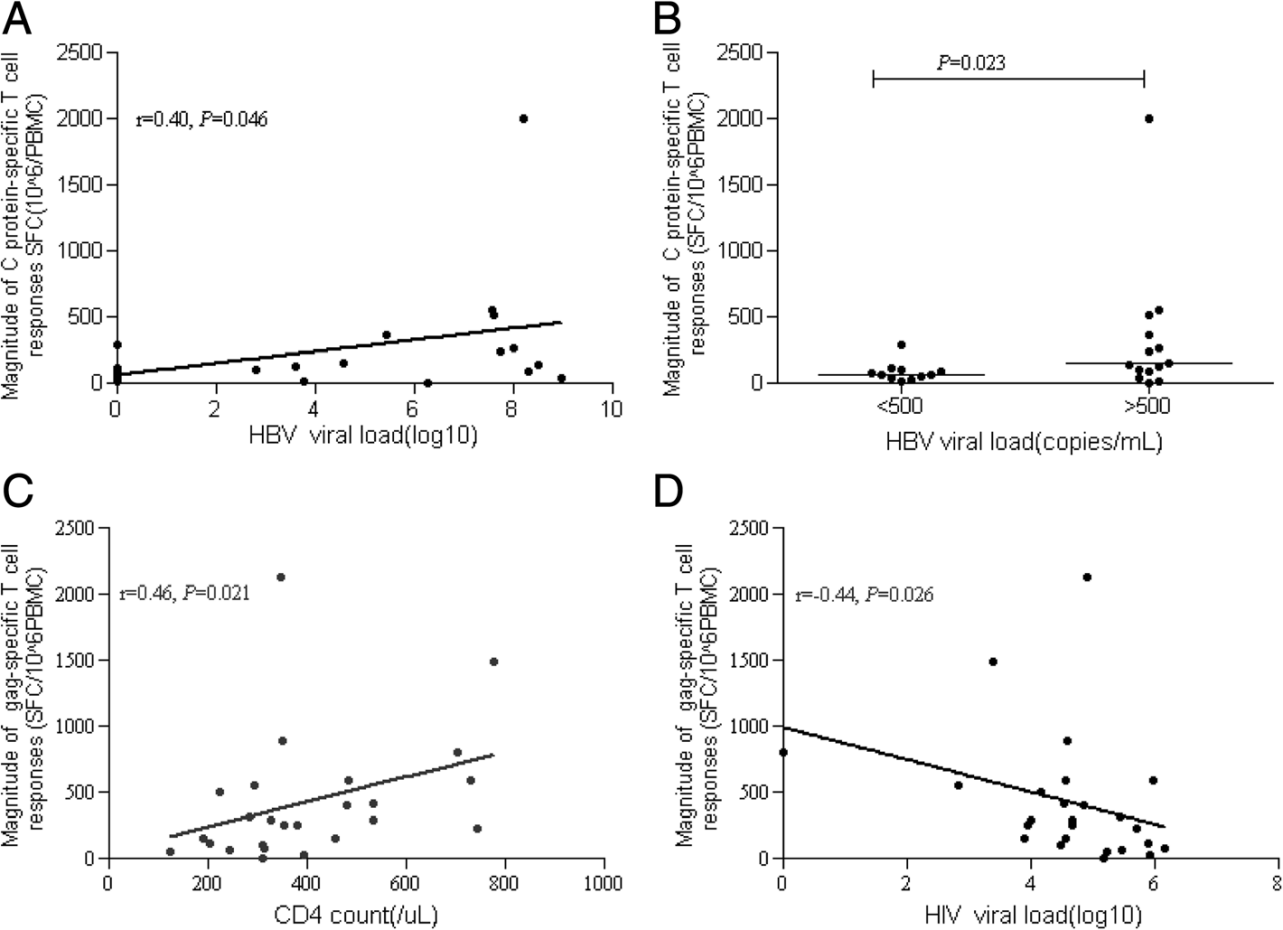
**Correlation of various HBV and HIV associated variables. (A)** Correlation of HBV viral load and HBV-specific T-cell responses elicited by chronic HBV patients co-infected with HIV. **(B)** Comparison of HBV-specific T-cell responses induced by HBV-infected patients with HIV coinfection grouped by HBV viral load (<500 copies/mL or >500 copies/mL). The relation of CD4 count **(C)** and HIV viral load **(D)** on gag-specific T-cell responses elicited by chronic HBV patients co-infected with HIV.

To analyze whether HBV viral load could affect the magnitude of HBV-specific T-cell responses in co-infected, chronic HBV patients, 25 subjects were divided into two groups according to the lower limit of the HBV viral load (lower detection limit of 500 copies/mL). As shown in Figure 2B, the magnitude of C protein-specific T-cell responses was significantly greater in subjects with an HBV viral load >500 copies/mL compared to subjects with an HBV viral load <500 copies/mL (p = 0.023). This difference was not observed in chronic HBV patients without HIV coinfection.

The relationship between HBV viral load and the magnitude of HBV-specific T-cell responses was also analyzed in the 25 co-infected, chronic HBV patients. As shown in Figure 2A, the C protein-specific T-cell responses were significantly positively correlated with HBV viral load (r_s_ = 0.40, p = 0.046). However, the magnitude of the HBV-specific T-cell response was not correlated with CD4^+^ T cell count or HIV viral load. The magnitude of gag-specific T-cell response was positively correlated with CD4^+^ T cell count (r_s_ = 0.46, p = 0.021; Figure 2C), but negatively correlated with HIV viral load (r_s_ = −0.44, p = 0.026; Figure 2D). Gag-specific T cell responses were significantly lower in co-infected, chronic HBV patients with an HIV viral load >5(log_10_) than in co-infected, chronic HBV patients with an HIV viral load <5(log_10_) (p = 0.005), though the difference in HBV-specific T-cell response was insignificant between these two groups. These data indicate that HBV-specific T-cell responses are higher in patients with higher HBV viral loads, but gag-specific T-cell responses are weaker in patients with lower CD4^+^ counts and higher HIV viral loads.

However, HBV-specific T-cell responses were not related to HBV viral load in mono-infected, chronic HBV patients.

### Relationship of HBV-specific T-cell responses to the presence of HBeAg

**Figure 3:**
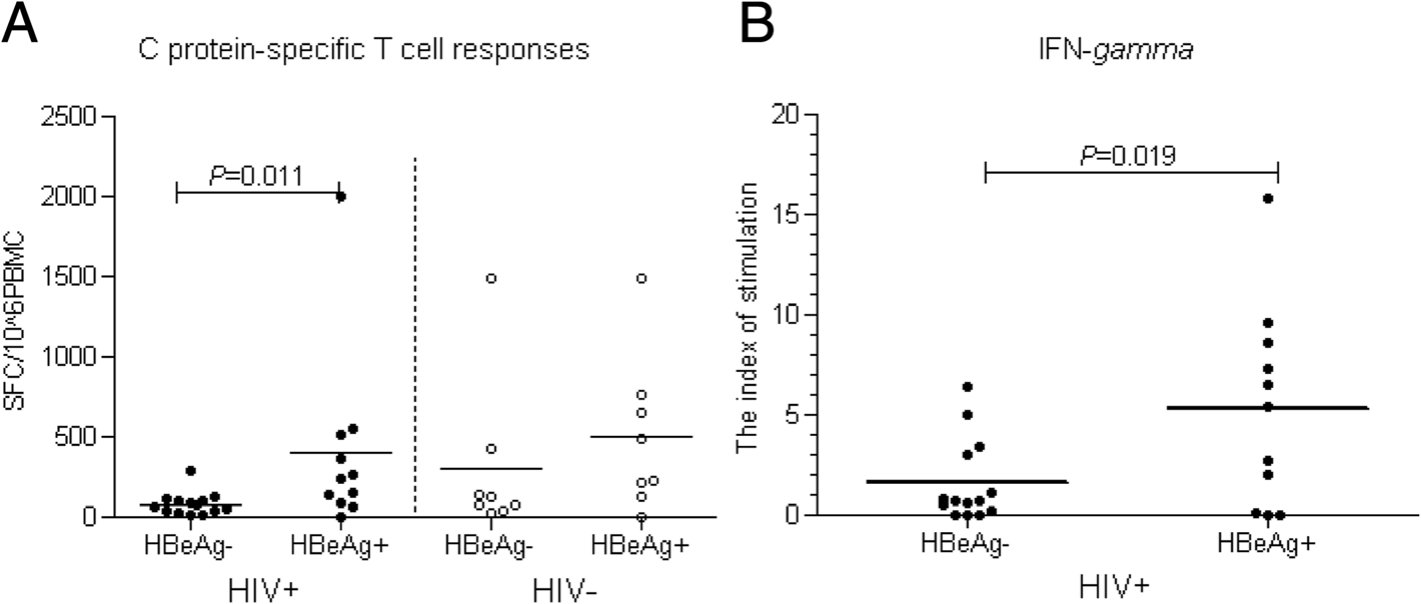
**Comparison of HBV-specific cell responses in the presence or absence of HBeAg. (A)** C protein-specific T-cell responses elicited in ELISPOT assay; **(B)** Cytokine IFN-Y responses in cultured supernatants of PBMCs stimulated by C protein. HBeAg(−): HBeAg-negative subjects; HBeAg(+): HBeAg-positive subjects; HIV+: HIV co-infected; HIV-: not co-infected with HIV.

HBV-specific T-cell responses to the presence of HBeAg were examined in co-infected, chronic HBV patients since HBeAg is an immunotoleragen [8]. Subjects were divided into HBeAg negative and HBeAg positive groups. The magnitude of the C protein-specific T-cell response was no different in HBeAg-positive, mono-infected, chronic HBV patients than in HBeAg-negative, mono-infected, chronic HBV patients (p > 0.05; Figure 3A right). In contrast, the magnitude of the C protein-specific T-cell response was significantly greater in HBeAg-positive, co-infected, chronic HBV patients than in HBeAg-negative, co-infected, chronic HBV patients (p = 0.011; Figure 3A left).

### Cytokine responses in cultured supernatants of PBMCs from co-infected, chronic HBV patients

To further ascertain whether HBeAg-positive, co-infected, chronic HBV patients have higher cytokine productivity compared to HBeAg-negative, co-infected, chronic patients, the levels of IL-2, IL-4, IL-6, IL-8, IL-10, GM-CSF, TNF-α, and IFN-γ secreted by PBMCs, stimulated with recombinant C protein, were measured in cultured supernatant. The level of IFN-γ secreted by HBeAg-positive patients was significantly higher than that secreted by HBeAg-negative patients (92.09[0.02 ~ 186.21]pg/mL vs. 28.33[0.02 ~ 80.22]pg/mL, respectively; p = 0.031). Moreover, the stimulation in index for cytokine IFN-γ was significantly higher for HBeAg-positive patients than for HBeAg-negative patients (p = 0.019; Figure 3B). However, there were no significant differences in stimulation indices for IL-2, IL-4, IL-6, IL-8, IL-10, GM-CSF, or TNF-α among the two subgroups.

### In the longitudinal study, HBV-specific T-cell responses were lower in patients with chronic HBV infection with disease progression

**Figure 4:**
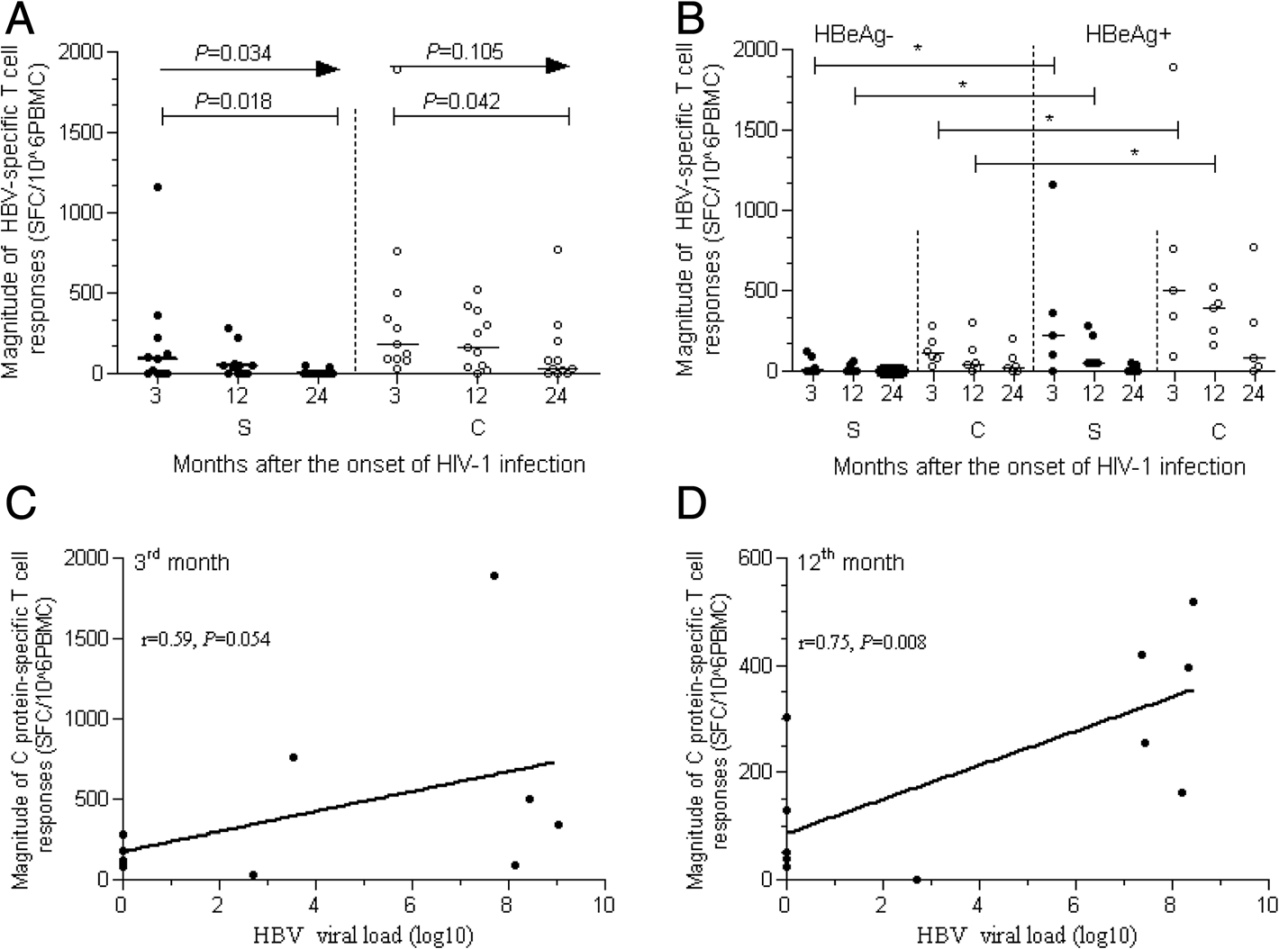
**HBV-specific T-cell responses induced in co-infected, chronic HBV patients followed up 3, 12, and 24 months after the onset of acute HIV-1 infection. (A)** the difference of HBV-specific T-cell responses elicited at different time points; **(B)** comparison of HBV-specific T-cell responses in the presence or absence of HBeAg; **(C)** correlation between C protein-specific T-cell responses and HBV viral load 3 months after the onset of acute HIV-1 infection; **(D)** correlation between C protein-specific T-cell responses and HBV viral load 12 months after the onset of acute HIV-1 infection; *p < 0.05 (Figure 4B); HBeAg^-^: HBeAg negative; HBeAg^+^: HBeAg positive.

The magnitude of HBV-specific T-cell responses was analyzed in 11 subjects that had been diagnosed as having acute HIV-1 infection and chronic HBV infection concurrently. As shown in Figure 4, S protein- and C protein-specific T-cell responses decreased as the length of follow-up increased (p = 0.034, for S protein; p = 0.105, for C protein; Figure 4A). S protein- and C protein-specific T-cell responses were significantly higher at 3 months after HIV infection than at 24 months after HIV infection (all p < 0.05; Figure 4A). Furthermore, the magnitude of C protein-specific T-cell responses was positively correlated with the levels of HBV DNA at 3 and 12 months post-HIV infection (r_s_ = 0.59, p = 0.054; Figure 4C and r_s_ = 0.75, p = 0.008; Figure 4D; respectively). Given the effects of HBeAg as an immunotoleragen, the relationship of the presence or absence of HBeAg to the HBV-specific T-cell response was also investigated. We found that the magnitude of S and C protein-specific T-cell responses in HBeAg-positive individuals was significantly higher than in HBeAg-negative patients at 3 and 12 months after HIV infection (all p < 0.05; Figure 4B), however there was no difference between HBeAg-positive and -negative patients at 24 months after HIV infection.

### HBV-specific T-cell responses, stimulated by S and C proteins, are mediated by CD4^+^ and CD8^+^ T cells

To identify the specific T-cell responses induced by a stimulus, CD4^+^ and CD8^+^ cells were isolated from PBMCs and an ELISPOT assay was performed. CD4^+^ T cells induced approximately 70-80% of the HBV-specific T-cell responses and CD8^+^ cells induced approximately 20-30% of the HBV-specific T-cell responses. In contrast, CD8^+^ cells induced about 96% of the gag-specific T-cell responses, but CD4^+^ cells induced very little of the gag-specific T-cell responses.

## Discussion

In the cross-sectional study, HBV-specific T-cell responses in chronic HBV patients, co-infected with HIV, were described. Specific T-cell responses, induced by S protein, were lower than specific T-cell responses induced by C protein regardless of whether the subject had an HIV coinfection. This is consistent with the previous studies that showed that chronic HBV infection is generally associated with a narrow HBV-specific response, with the dominant responses often directing the core protein [10,11]. Overall, in the co-infected, chronic HBV patients, HBV-specific T-cell responses were weaker than in the mono-infected patients, though the difference was not significant. Mono-infected, chronic HBV patients had significantly weaker C protein-specific T-cell responses compared to those without HIV who were previously infected with HBV (p = 0.021, data not shown). However, the magnitude of C protein-specific T-cell response was comparable in co-infected, chronic HBV patients and individuals that had HIV who were previously infected with HBV (p > 0.05, Figure 1). HBeAg has been described as an immunologic toleragen, however it is not clear whether HBeAg has suppressive effects on the immune response to HBV in the presence of HIV-1. We found no significant difference in C protein-specific T-cell responses between HBeAg-positive patients with HIV-1 and HBeAg-positive patients without HIV-1. However, co-infected, HBeAg-positive patients had greater HBV-specific T-cell responses than co-infected, HBeAg-negative patients. Furthermore, there was no relationship between C protein-specific T-cell responses and the level of HIV RNA or CD4 count in co-infected, HBeAg-positive patients (data not shown). Based on these observations, it appears that HBeAg does not have suppressive effects on HBV-specific T-cell responses in the presence of HIV-1.

The magnitude of HBV-specific T-cell responses was positively correlated with the level of HBV DNA in co-infected, chronic HBV patients. However, this was not the case in mono-infected, chronic HBV patients. Similar results were observed were in the longitudinal study. Therefore, we hypothesize that the greater HBV-specific T-cell responses seen in chronic HBV patients with HIV-1 may be related to higher levels of HBV DNA. This observation is contradictory to previous studies that indicating that a reduction in HBV viral load may lead to a loss of HBV anergy in chronic HBV patients [12,13]. One possible explanation for this discrepancy is that the anergy to HBV seen in our study may have been partly activated by HIV-1 co-infection. An alternative explanation is that HBV-specific T cells may migrate from the liver to the periphery in the presence of HIV-1, as observed with antiviral HBV therapy [14], however the precise reason(s) and mechanism(s) need to be explored.

We found that HBeAg-positive patients with higher levels of plasma HBV viral load, had greater HBV-specific T-cell responses than HBeAg-negative patients with no or very low levels of HBV viral load. Conversely, in mono-infected, chronic HBV patients, there was no difference in HBV-specific T-cell responses between HBeAg-positive and -negative patients (Figure 3), despite significant differences in HBV DNA levels. This finding was further confirmed by data showing that cultured supernatants from HBeAg-positive patients contained more specific-IFN-γ than that from HBeAg-negative patients, although there was no difference in levels of IL-2, IL-4, IL-6, IL-8, IL-10, GM-CSF, or TNF-α between HBeAg-positive and -negative patients. Additionally, we found that HBeAg-positive individuals had stronger HBV-specific T-cell responses than HBeAg-negative patients at 3 and 12 months after the onset of acute HIV-1 infection. These findings are contradictory to the Chang et al. study which reported that the magnitude of the HBV-specific IFN-γ –positive CD8^+^ T-cell response was not significantly different between HBeAg-positive and -negative patients in HIV/HBV co-infected, ART naïve patients [15]. The distinct immunogenicity of stimulus antigens used in the two studies may have led to the different outcomes. Chang et al. [15] used a 15 amino acid peptide pool of four proteins, however 14–22 amino acid peptides are typically used to induce either CD4^+^ or CD8^+^ T-cell responses [16] and mainly induce a CD8^+^ T-cell response [17]. Recombinant whole protein can be used to detect CD4-mediated responses and a limited CD8-mediated response [16,18]. In this study, HBV-specific T-cell responses were not only mediated by CD4^+^ T-cells but also by CD8^+^ T-cells, as described by Kalyuzhny et al. [16], when recombinant protein was used as the stimulus. An alternative explanation is that the different disease statuses may have contributed to the different outcomes. Patients in the present study were considered to have mild disease, had normal ALT levels for at least two years and higher CD4 counts, however the patients in the Chang study had more severe disease with higher ALT levels and lower CD4 counts.

In chronic HBV infection, ALT is considered a marker of hepatic necrosis and inflammation. A lower ALT level reflects less hepatocyte destruction, which contributes to weak HBV-specific T-cell responses [19,20]. In the current study, the co-infected, chronic HBV patients had normal ALT levels during the two years of observation. One of possible explanation is that the HBV-specific T-cell responses were weak. In fact, C protein-specific T-cell responses in co-infected, chronic HBV patients were much weaker than in individuals who had been previously infected HBV, but did not have HIV (p = 0.005, data not shown). Moreover, the impaired quality of HBV-specific CD8^+^ T-cells [15] and concurrent loss of CD4^+^ cells in co-infected, chronic HBV patients could result in HBV virus not being cleared, thus prolonging the chronic HBV infection. Nevertheless, the biological significance of these findings and their association with normal liver function and clinical progression remains to be determined.

Previous studies have reported that individuals recover from HBV infection by acquiring specific immunity to HBV. In the current cross-sectional study, we also found that individuals who had been previously infected with HBV (without HIV co-infection) had stronger HBV specific T-cell responses. However, HBV-specific T-cell responses in individuals previously infected with HBV were exceptionally lower in the presence of HIV-1 (Figure 1); the precise reason(s) and/or mechanism(s) for this need to be analyzed. As observed in the cross-sectional study, CD4^+^ cells mainly induced C protein-specific T-cell responses. Chang et al. [21] reported that HBV-specific CD4^+^ T cells were reduced in HIV/HBV co-infected, chronic patients that received HBV antiretroviral therapy. Thus, we believe that the loss of CD4^+^ T cells could lead to a significantly decreased CD4-specific immune response to HBV. On the other hand, since CD4^+^ T- cells are required to sustain CD8^+^ cytotoxic T-cell responses during chronic viral infection [22-24], the depletion of CD4^+^ T-cell function may cause a reduction in HBV-specific CD8^+^ T-cell responses. Indeed, Lascar et al. [25]. reported a reduction in human leukocyte antigen (HLA)-A2-restricted HBV-specific (core 18–27) CD8^+^ T-cell response in previously infected HBV subjects who were HIV-positive compared with previously infected HBV subjects who were HIV-negative. Therefore, it is thought that the decrease in both HBV-specific CD4^+^ and CD8^+^ T-cell responses may account for the weak HBV-specific T-cell responses in individuals with HIV who were previously infected with HBV. Furthermore, the shortage of HBV antigen may be another factor leading to the lower HBV-specific T cell responses in individuals with HIV who had been previously infected with HBV. The reduction in HBV-specific T-cell responses could account for the relatively high prevalence of occult Hepatitis B in HIV-infected patients, especially after withdrawal of HAART [26]. Although the level of plasma HBV DNA was undetectable in the current study in individuals with HIV that had been previously infected with HBV, continuous visits would be required to monitor clinical progression.

There were limitations in the present study. First, we did not know whether co-infected, chronic HBV patients in the cross-sectional study acquired HBV infection prior to HIV infection. However, based on previous epidemiological studies of HBV infection in China, it is highly likely that most of the individuals in cross-sectional study acquired HBV at birth or in early childhood. Patients with HIV in this study were infected as an adult via the route of homosexual contact. Thus, it is reasonable to assume that the individuals in the cross-sectional study were infected with HIV subsequent to HBV. Secondly, we were not able to collect accurate data regarding the time of HBV acquisition in cross-sectional study and could not take into account potential differences in the duration of HBV infection among the mono-and co-infected patients.

Overall, the HBV-specific T-cell responses in co-infected, chronic HBV patients decreased with disease progression and were positively correlated with HBV viral load. The HBV-specific T-cell responses significantly lower in individuals with HIV who had been previously infected with HBV compared with individuals not co-infected with HIV. Although almost all co-infected, chronic HBV patients had normal liver function in this study, longer follow-up studies of subjects with HIV/HBV co-infections are required to determine the kinetic changes in HBV-specific immune responses and to monitor the disease progression in order to come up with effective approaches to manage those with HIV/HBV co-infections.

## Methods

### Subjects

In the current study, 39 patients infected with HIV-1 were recruited from Beijing Youan Hospital, Capital Medical University, China, to participate in a cross-sectional study. A total of 25 patients had chronic HBV and 14 had been previously infected with HBV. Patients had no history of treatments for either HIV or HBV. The control group was comprised of 32 individuals that were not infected with HIV-1; 16 subjects had chronic HBV and 16 subjects had been previously infected with HBV. All subjects were older than 18 years of age and were negative for hepatitis C virus antibody. In the chronic HBV patients, HBsAg was positive for >6 months prior to analysis. Exclusion criteria included hepatocellular carcinoma, autoimmune liver disease, and liver cirrhosis. Patients that had previous HBV infection were negative for HBsAg, positive for HBsAb, either positive or negative for HBeAb, and positive for HBcAb.

A total of 11 individuals with acute HIV-1 and chronic HBV infection, who had been HBsAg positive for >5 years, were enrolled in a longitudinal study. Follow-up was done in these patients at 3, 12, and 24 months after the onset of acute HIV infection. Signed consent was obtained from all subjects with the approval of the institutional ethics committee and the study was in compliance with the Declaration of Helsinki.

### Human interferon (IFN)-γ ELISPOT assay

Peripheral whole blood was obtained from all subjects and peripheral blood mononuclear cells (PBMCs) were isolated by density gradient centrifugation with Ficoll Lymphoprep (Axis –Shield PoC AS, Oslo, Norway). Isolated PBMCs were kept in liquid nitrogen. After thawing, PBMCs were incubated at 37°C in 5% CO_2_ overnight. PBMCs (2.0 × 10^5^) were pulsed with 4 μg/mL recombinant Hepatitis B surface protein and recombinant Hepatitis B core protein (ARP, American Research Products, Inc. ™, USA), respectively, and were tested using a standard Human IFN-γ ELISPOT assay, in duplicate wells, as described previously [27]. PBMCs (2.0 × 10^5^), from subjects with HIV infection, were pulsed with 1 μg/mL gag peptide pool (comprised of 61 peptides covering the whole gag protein). In brief, assays were carried out in 96-well MultiScreen filter plates (Millipore) coated with 15 μg/mL anti–IFN-γ mAb 1-DIK (Mabtech AB, Nacka, Sweden). Three μg/mL phytohemagglutinin (PHA) (final concentration) was used as a positive control and the negative control wells were stimulated with RPMI 1640 containing 10% FCS. Plates were incubated at 37°C in 5% CO_2_ for 16 h. Spot enumeration was performed with an ELISPOT reader system (Antai Yongxin Medical Technology, Beijing, China). To quantify antigen-specific responses, mean spots of the control wells were subtracted from the positive wells, and results were expressed as spot-forming cells (SFC) per 10^6^ PBMCs. Responses were considered positive if results were at least twice the mean of the duplicate negative control wells and >30 SFCs/10^6^ PBMCs.

### CD4^+^ and CD8^+^ T-cell ELISPOT assay

CD4^+^ or CD8^+^ T cells were isolated from PBMCs using the Dynabeads CD4 kit or Dynabeads CD8 kit (Invitrogen Dynal As, Oslo, Norway), according to the manufacturer’s instructions. 2.0 × 10^5^ CD4^+^ or CD8^+^ T-cells were pulsed with 4 μg/mL recombinant Hepatitis B surface protein, recombinant Hepatitis B core protein (ARP, American Research Products, Inc.™, USA), or with 1 μg/mL of gag peptide pool, and were tested using the standard human IFN-γ ELISPOT assay in duplicate wells as above described.

### Cytokine responses in cultured supernatants

Samples of PBMC supernatants, from chronic HBV patients co-infected with HIV, were stimulated with 4 ug/mL recombinant C protein. Cultured supernatants were harvested after 2.0 × 10^5^ PBMCs were stimulated, overnight. The negative control well was not stimulated with recombinant C protein but with RPMI 1640 containing 10% FCS. Levels of cytokines in the cultured supernatants, including IL-2, IL-4, IL-6, IL-8, IL-10, GM-CSF, TNF-α, and IFN-γ, were detected using the Bio-Plex Pro™ Human Cytokine Grp I Panel 8-plex kit from Bio-Rad® (magnetic beads, Luminex®, USA). The outcome was determined using the index of stimulation (IS) to display the cytokine level of stimulated well. The index of stimulation was taken as the cytokine level of the stimulated well minus the cytokine level of un-stimulated well divided by the cytokine level of un-stimulated well.

### HBV DNA and HIV-1 RNA quantification

HBV DNA was quantified using a domestic HBV DNA assay kit (KeHua Biological Technological Corporation, Beijing, China), according to the manufacturer’s instructions (lower limit of detection, 500 copies/mL). HIV RNA was quantified using the Roche RT-PCR assay according to the manufacturer’s instructions (Easy Q; Roche; lower limit of detection, 40 IU/mL).

### Graphing and statistical analysis

Graphpad Prism 5 software was used to graph and analyze the data and P < 0.05 was regarded as statistically significant. Proportion of subjects by gender was determined using Fisher’s exact test. Comparison of age, HBV DNA, CD4^+^ T-cell count, and the magnitude and frequency of specific T-cell responses between the two groups was determined using the Student’s t test for parameter variables or the Mann–Whitney U test for non-parameter variables. The correlation between HBV-specific T-cell responses and HBV DNA or CD4^+^ T-cell count was determined using Spearman’s correlation analysis.

## Competing interests

The authors declare that they have no competing interests.

## Authors’ contributions

ZX designed the study, carried out all experiments, performed the statistical analysis and drafted the manuscript. XHQ participated in screening and observing the clinical cases and helped to draft the manuscript. FX carried out part of experiments and screened the clinical cases. ZHP carried out part of experiments. WY carried out part of experiments. YHP conceived of the study, and participated in its design and coordination and helped to draft the manuscript. All authors read and approved the final manuscript.

## Funding sources

This study was founded by Major Project of Beijing Municipal Science and Technology Committee (D09050703590904; D09050703590901), and the National 12th Five-Year Major Projects of China (2012ZX10001-008), and Youan Liver disease and AIDS Foundation, China Primary Health Care Foundation(BJYAH-2011-024).
